# Rifampicin-resistant tuberculosis in Iran: A systematic review and meta-analysis

**DOI:** 10.22038/ijbms.2021.47360.10901

**Published:** 2021-06

**Authors:** Farhad Bahraminia, Taher Azimi, Moein Zangiabadian, Mohammad Javad Nasiri, Mehdi Goudarzi, Masoud Dadashi, Abbas Ali Imani Fooladi

**Affiliations:** 1 Applied Microbiology Research Center, Systems Biology and Poisonings Institute, Baqiyatallah University of Medical Sciences, Tehran, Iran; 2 Department of Pathobiology, School of Public Health, Tehran University of Medical Sciences, Tehran, Iran; 3 Student Research Committee, School of Medicine, Shahid Beheshti University of Medical Sciences, Tehran, Iran; 4 Department of Microbiology, School of Medicine, Shahid Beheshti University of Medical Sciences, Tehran, Iran; 5 Department of Microbiology, School of Medicine, Alborz University of Medical Sciences, Karaj, Iran

**Keywords:** Drug resistance, Iran, Rifampicin, Tuberculosis, Xpert MTB/RIF assay

## Introduction

Multidrug-resistant tuberculosis (MDR-TB), poses a global threat to TB control programs, especially in developing countries (1). In 2019, among the 81 million people in Iran, there was an estimated TB incidence of 13 per 100,000 population (1). Estimated Iran MDR/ rifampicin (RIF)-resistant TB rates were 1.3% among new cases and 8.3% in retreatment cases (1). Patients with RIF-resistant TB, often seen as a proxy for MDR-TB, require treatment regimens that are longer, less effective, and less accessible than first-line regimens (2-8). The low numbers of well-equipped laboratories for drug susceptibility testing (DST) in Iran, make the diagnosis of RIF-resistance challenging in the country (6, 9, 10). As a result, RIF-resistant-TB, very often remains undetected, leading to further spread of drug-resistant TB and worse TB treatment outcomes (11-15). Given that RIF-resistant TB is among the major challenges for national TB control programs (NTP), identification of RIF-resistant TB resistance among *Mycobacterium tuberculosis* isolates could help us to better advance treatment achievement. Although some studies have investigated the prevalence of RIF-resistance in Iran, a comprehensive analysis has not yet been reported. In this study, we aimed to assess the frequency of RIF-resistance in *M. tuberculosis* isolates in Iran, using a systematic review and meta-analysis.

## Materials and Methods

***Search strategy***

Pubmed/Medline, Embase, Web of Science, and Scopus from January 1, 1980, to January 1, 2020, were screened for English articles that contained the terms “tuberculosis”, “rifampicin”, and “Iran”. Details of strategies used in Pubmed/Medline are given in Table S1 in the Appendix. Articles in Persian were also searched in the Iranian databases (SID [www.sid.ir] and Magiran [www.Magiran.com]) with similar strategies and related Persian keywords. We performed a systematic review and meta-analysis of the literature following PRISMA guidelines (16). 

***Study selection***

All articles identified by the initial search were reviewed independently by two reviewers (FB and MJN) for relevance, with disagreements mediated by a third author (AAIF). The same reviewers also double reviewed all full-text articles. Studies were selected for inclusion if they met the following criteria: 1) presented original data; 2) provided the primary data on the total number of patients with TB, as well as the number of those with RIF-resistance; and 3) used the standard phenotypic DST method as recommended by WHO/CDC (17, 18). Data from studies evaluating molecular drug susceptibility tests were also included if the results were verified by DNA sequencing. Studies with unrepresentative samples of the general population of TB as well as insufficient information about patients’ characteristics were excluded. 

***Data extraction***

Two reviewers (FB and MJN) performed double data extraction and entry using Microsoft Excel. A third reviewer (AAIF) judged any discrepancies between the two reviewers. From each study, study location, design, age, year, the total number of TB patients, number of RIF-resistance, as well as, when available, status of HIV, and history of the previous TB among participants were extracted. All data were extracted and compiled using the MS Excel software package (Microsoft, Redmond, WA, USA).

In the text, the term “new cases” refers to patients with TB who have never received anti-TB drugs. The term “previously treated cases” or “history of treatment” is used to refer to patients who had previously received anti-TB drugs. “RIF mono resistance” was used to define the resistance to only RIF. “RIF any resistance” referred to resistance to any kind of RIF resistance regardless of mono-resistance or multi-drug resistance (resistance to at least isoniazid and rifampicin).

***Quality assessment***

Two authors (FB and MJN) applied the Joanna Briggs Institute quality assessment tool for cross-sectional studies to assess the risk of bias for each study. They independently evaluated the components of the scale as “Yes”, “No”, “Unclear” or “Not Applicable”. This was used to guide the overall rating for the quality of each study as “Good”, or “Poor”. In case of disagreement, a consensus opinion was reached.

***Meta-analysis***

Statistical analyses were performed with STATA (version 14, IC; Stata Corporation, College Station, TX, USA). The pooled frequency of RIF-resistance among patients with confirmed TB was assessed by the random-effects model. Heterogeneity across studies was estimated by calculating the I^2 ^statistic. A *P-*value of less than 0.05 indicated that heterogeneity among the group of studies being analyzed was significant. To explore sources of studies’ heterogeneity, we did meta-regression and subgroup analysis. Publication bias was assessed statistically by using Begg’s tests (*P*<0.05 was considered indicative of statistically significant publication bias). 

## Results

**Figure 1:**
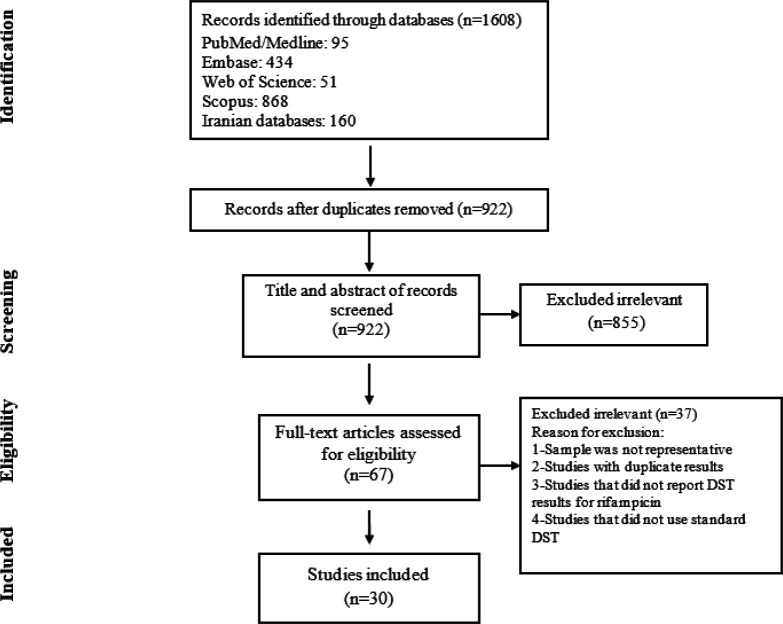
Flow chart of study selection for inclusion in the systematic review and meta-analysis

The results of the literature search are displayed in Figure 1. Our initial search yielded 1608 studies. Of these, 67 were referred for full-text assessment, and 30 cross-sectional studies met the inclusion criteria and were selected for inclusion in the qualitative synthesis and meta-analysis (10, 19-47). Table 1 provides information on each of the included studies. Studies were conducted in different regions of Iran: Tehran was the most frequently represented city with 13 studies. In all included studies, conventional DST was performed by the standard method according to the WHO or CDC guidelines. The sample size ranged from 31 to 1242 individuals enrolled per study. A total of 8215 patients with TB were included in the meta-analysis. Five studies reported RIF mono-resistance for a total of 3205 TB cases. Although we sought to extract data on HIV infection and previous TB treatment, most studies did not provide sufficient information. Data on previous TB treatment was provided by only five of the 30 included studies and HIV infection by one. 

***Quality assessment***

All included studies were rated as “Good” by both assessors, representing a low risk of bias.

***Frequency of RIF-resistance among patients with TB***

**Figure 2:**
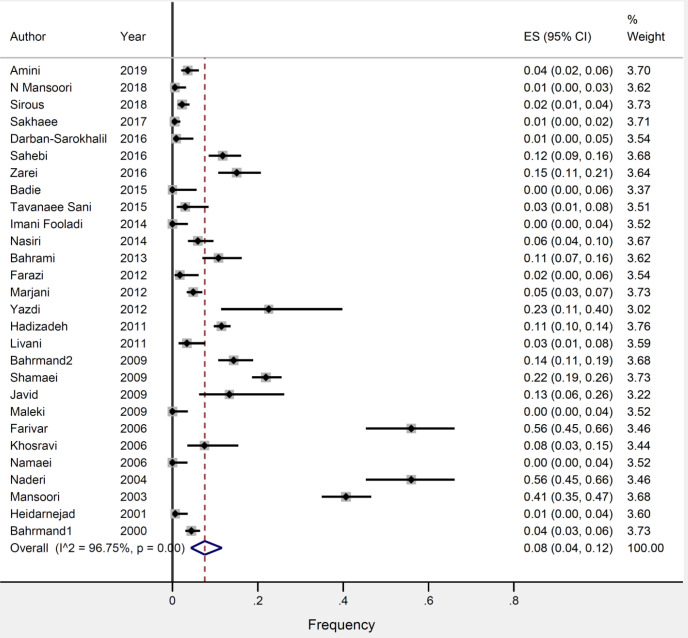
Frequency of RIF-resistance among patients with confirmed TB

RIF: Rifampicin; TB: tuberculosis

As shown in Figure 2, the overall frequency of RIF-resistance among all patients with TB was 8.0% (95% CI 4.0–12.0). We found a high degree of heterogeneity in the results across the included studies (I2=96%, *P*=0.00). Based on meta-regression, the number of RIF-resistances per study resulted in a significant source of heterogeneity in the current study (*P*-value= 0.03). As per Begg’s (*P*=0.1) test, there was no evidence of publication bias. 

***Subgroup analysis***

Table 2 shows the subgroup analysis of the studies based on the type of RIF-resistance, and history of TB treatment. RIF-resistance was significantly higher among previously treated patients compared to new patients (4% vs 36%). 

**Table 1:** Characteristics of the included studies investigating the frequency of RIF-resistance among patients with confirmed TB

**First author**	**Published** **time**	**Enrollment** **time**	**Location **	**Mean age**	**Total No. of TB patients**	**Total No. of RIF-resistance**	**Type of patients**	**DST method**
Amini	2019	2015-2017	Multicenter	Adult	334	12	New and retreatment case	WHO standard conventional DST
N Mansoori	2018	2014-2015	Golestan	50	176	1	New cases	WHO standard conventional DST
Sirous	2018	2015-2017	Ahvaz	NR	487	11	NR	WHO standard conventional DST
Sakhaee	2017	2013-2016	Tehran	NR	395	2	NR	CDC standard conventional DST
Darban-Sarokhalil	2016	NR	Tehran	Adult	112	1	New cases	WHO standard conventional DST
Sahebi	2016	2011-2013	Multicenter	52	280	33	New and retreatment case	WHO standard conventional DST
Zarei	2016	2012-2014	Shiraz	48	199	30	NR	WHO standard conventional DST
Badie	2015	NR	Ahvaz	Adult	64	0	NR	WHO standard conventional DST
Tavanaee Sani	2015	2012-2013	Mashhad	NR	100	3	New and retreatment case	WHO standard conventional DST
Imani Fooladi	2014	2009-2011	Tehran	Adult	103	0	NR	WHO standard conventional DST
Nasiri	2014	2010-2012	Multicenter	45	252	15	New cases	WHO standard conventional DST
Velayati	2014	2010-2011	Tehran	47	1242	NR	New and retreatment case	WHO standard conventional DST
Bahrami	2013	2010-2012	Tehran	Adult	176	19	NR	WHO standard conventional DST
Farazi	2012	2005-2010	Arak	52	115	2	New and retreatment case	WHO standard conventional DST
Marjani	2012	2003-2008	Tehran	51	554	27	New and retreatment case	WHO standard conventional DST
Yazdi	2012	2009-2010	Yazd	NR	31	7	New cases	WHO standard conventional DST
Hadizadeh	2011	2006-2009	Tehran	NR	1027	118	NR	WHO standard conventional DST
Livani	2011	2009-2010	Golestan	54	148	5	New and retreatment case	MGIT
Bahrmand2	2009	2005-2006	Tehran	Adult	286	41	NR	CDC standard conventional DST
Shamaei	2009	2000-2003	Tehran	45.4	548	120	New and retreatment case	WHO standard conventional DST
Javid	2009	2007-2008	Golestan	NR	45	6	New cases	WHO standard conventional DST
Maleki	2009	2007-2008	Tabriz	NR	103	0	NR	WHO standard conventional DST
Farivar	2006	2001-2003	Zahedan	Adult	84	47	New and retreatment case	WHO standard conventional DST
Khosravi	2006	NR	Ahvaz	Adult	80	6	NR	WHO standard conventional DST
Namaei	2006	2001-2002	Mashhad	56.6	105	0	New cases	WHO standard conventional DST
Naderi	2004	2001-2002	Zahedan	NR	84	47	New and retreatment case	WHO standard conventional DST
Mansoori	2003	1996-2000	Tehran	37	273	111	New and retreatment case	WHO standard conventional DST
Heidarnejad	2001	NR	Tabriz	44	155	1	New and retreatment case	WHO standard conventional DST
Moniri	2001	1998-2000	Kashan	75	94	NR	NR	WHO standard conventional DST
Bahrmand1	2000	1998-1999	Tehran	Adult	563	25	New	WHO standard conventional DST

RIF: Rifampicin; TB: tuberculosis

**Table 2:** Pooled frequency of RIF-resistance among subgroups of studies

**Subgroups **	**No. of study**	**Frequency (95 % CI)**	**Heterogeneity**	
			***P*** **-Value**	**I2 (%)**
Type of RIF-resistance Any resistance Mono resistance	28 (6879 TB cases) 5 (3205 TB cases)	8.0 (4.0-12.0) 5.0 (0.0-12.0)	0.00 0.00	96 100
History of treatment New cases Previously treated cases	10 (1904 TB cases) 5 (383 TB cases)	4.0 (2.0-8.0) 36.0 (2.0-82.0)	0.00 0.00	79 100

RIF: Rifampicin

## Discussion

 In the present study, the pooled frequency of RIF-resistant TB in all TB cases was found to be 8.0%. Our sub-group analysis also showed that 4.0% of newly diagnosed cases and 36.0% of previously-treated TB patients from different settings in Iran were RIF-resistant. The prevalence of RIF-resistant TB among new cases observed in this study is above the current WHO estimates of drug resistance for Iran (1). This suggests that the burden of RIF-resistance in new patients with TB may be underestimated and better programmatic strategies are needed. 

Furthermore, several other studies reported quite a varied frequency of RIF-resistant TB in the different countries in the Middle East Region. The prevalence of RIF-resistant TB in this study compared to previous studies in Iraq (12.6%), Egypt (1.9%), Turkey (1%), Saudi Arabia (1%), and Kuwait (0.2%) (48). The variation of RIF-resistant-TB across the country might be related to geographical variation, study setting, differences in patient selection, sample size, method of diagnosis, and TB control practice.

Several countries in the world have adopted an algorithm placing Xpert MTB/RIF as the initial and diagnostic test for RIF-resistance (49-55). The results from the early programmatic implementation of Xpert MTB/RIF testing in nine countries indicated that testing with Xpert MTB/RIF can detect a large number of people with TB that routine services failed to detect (56). As more cases are rapidly detected and treated, there will be a reduction in transmission of primary drug resistance in the community. In Iran, due to limited resources, only a few TB laboratories use Xpert MTB/RIF for rapid diagnosis of TB and detection of drug resistance. Accordingly, in the current systematic review, all studies used conventional DST for investigating the drug-resistant pattern in patients infected with *M. tuberculosis*. 

We also indicated that near half of previously-treated TB patients in the current study were resistant to RIF (Table 3). This indicates that in Iran there may be high rates of acquired resistance to RIF. Failure of the appropriate treatment of TB patients is among the most common causes of the occurrence of drug resistance. This could be from the supply or quality of the drugs, possible inadequate drug intake by patients, and deficient infection control in hospitals (57, 58). Our results suggest that NTP needs to strengthen the management of drug-resistant TB, and patients previously treated for TB should be prioritized in case findings.

This review has some limitations. Not all regions in Iran had reported RIF-resistant TB, as such these were considered not fully representative. Another limitation was that not all necessary information, such as age, sex, ethnicity, and HIV, could be obtained from all included studies. Therefore, relevant stratified analyses could not be performed to find out more details of the related risk factors. 

## Conclusion

Our study showed that the frequency of RIF-resistance among patients with TB was 8.0%. Programmatic implementation of rapid DST such as the Xpert MTB/RIF assay as a primary diagnostic test for persons suspected of having a RIF-resistant TB would be helpful for control of the drug resistance. 

## Founding

This study was funded by a grant from National Institute for Medical Research Development (grant no: 976978).
